# ﻿Non-canonical telomeric motif TTAGGGGTGG in the true bug species *Geocorisdispar* Waga, 1839 (Heteroptera, Geocoridae)

**DOI:** 10.3897/compcytogen.19.156983

**Published:** 2025-07-16

**Authors:** Natalia Golub, Boris Anokhin, Valentina Kuznetsova

**Affiliations:** 1 Zoological Institute of the Russian Academy of Sciences, Universitetskaya Emb. 1, St. Petersburg 199034, Russia Zoological Institute of the Russian Academy of Sciences St. Petersburg Russia

**Keywords:** FISH, *Geocorisdispar*, Hemiptera, Lygaeoidea, Pentatomomorpha, (TTAGGGGTGG)_*n*_ telomeric sequence

## Abstract

We report on the results of a chromosomal study of the big-eyed true bug *Geocorisdispar* Waga, 1839 (Heteroptera: Geocoridae) aimed at obtaining data on its telomeres. Using fluorescence *in situ* hybridization (FISH), we have shown, that *G.dispar* has a non-canonical 10-bp telomeric motif TTAGGGGTGG. This is the first evidence of telomere structure in the family Geocoridae and the first finding of the telomeric sequence (TTAGGGGTGG)*_n_* in the large superfamily Lygaeoidea (infraorder Pentatomomorpha).

## ﻿Introduction

The pentanucleotide repetitive sequence (TTAGG)_*n*_, referred to as the “insect/arthropod-type” telomeric motif TTAGG, is the most phylogenetically widespread and presumably ancestral telomeric motif in insects and in arthropods in general (Sahara et al. 1999; Frydrychová et al. 2004; Vítková et al. 2005; Kuznetsova et al. 2020; Zhou et al. 2022). However, it is now well established that this motif has been repeatedly replaced by other short motifs or other DNA sequences throughout the course of insect evolution. These replacements include telomere-associated transposable elements or long nucleotide repeats ranging from 173 to 381 base pairs (Mravinac et al. 2011; Mason et al. 2016; Kuznetsova et al. 2020, 2025; Prušáková et al. 2021; Lukhtanov and Pazhenkova 2023; Stoianova et al. 2024). Most recently, several alternative short telomeric motifs, varying in length from one to 11 nucleotides (Lukhtanov and Pazhenkova 2023), have been identified in many insect species using Illumina short-read sequencing of genomes (Zhou et al. 2022) or analysis of chromosome-scale genome assemblies (Lukhtanov and Pazhenkova 2023; Kuznetsova et al. 2025; Lukhtanov 2025; Rico-Porras et al. 2025).

Despite the apparent progress in the study of insect telomeres, the telomere structure remains unknown in many species that have lost the canonical motif TTAGG. In the species-rich true bug suborder Heteroptera (order Hemiptera), telomeric sequences are now known in 40 species (i.e., no more than 0.1% of all described recent species of true bugs) from 30 genera and 13 families that have been examined by fluorescence *in situ* hybridization (FISH) and/or chromosome-level genome assembly analysis (Kuznetsova et al. 2012, 2025; Pita et al. 2016; Angus et al. 2017; Chirino et al. 2017; Grozeva et al. 2019; Gapon et al. 2021; Lukhtanov and Pazhenkova 2023; Stoianova et al. 2024). The species studied belong to three of the seven recognized infraorders (Štys and Kerzhner 1975), including the Nepomorpha, which represents an early branch of Heteroptera, as well as two evolutionarily advanced sister infraorders Cimicomorpha and Pentatomomorpha, which comprise the highly diversified clade Terheteroptera, the terminal group of true bugs (Weirauch et al. 2019). Current evidence suggests that true bugs exhibit heterogeneity in telomere structure, at least as shown by recent data obtained for Terheteroptera (see below). The canonical 5-bp “insect-type” motif TTAGG was only found (in the absence of any alternative motifs) in Nepomorpha, in both families studied: Belostomatidae and Nepidae (Kuznetsova et al. 2012; Angus et al. 2017; Chirino et al. 2017). Additionally, this motif was found in the family Reduviidae (Pita et al. 2016; Grozeva et al. 2019; Gapon et al. 2021; Kuznetsova et al. 2025), which is the sister lineage to the other families of Cimicomorpha (Schuh and Štys 1991; Schuh et al. 2009). In contrast, all other examined species of Cimicomorpha (from the families Anthocoridae, Cimicidae and Miridae) and all species of Pentatomomorpha analyzed (from the families Acanthosomatidae, Pentatomidae, Coreidae, Alydidae, Rhopalidae, Lygaeidae and Blissidae) exhibited the ten-letter motifs TTAGGGATGG, TTAGGGGTGG, and TTAGGGTGGT (Lukhtanov and Pazhenkova 2023; Stoianova et al. 2024; Kuznetsova et al. 2025). The first motif proved to be the most prevalent, appearing in both infraorders, while the latter two motifs were found exclusively in Pentatomomorpha: TTAGGGGTGG in Pentatomidae, Coreidae and Alydidae (in each family alongside TTAGGGATGG), and TTAGGGTGGT in the family Acanthosomatidae, where only one species, *Acanthosomahaemorrhoidale* (Linnaeus, 1758), has been studied (Lukhtanov and Pazhenkova 2023; Stoianova et al. 2024; Kuznetsova et al. 2025).

Our recent attempt to identify the telomeric motif in *Geocorisdispar* Waga, 1839, a representative of another family of Pentatomomorpha, the Geocoridae, using fluorescence *in situ* hybridization (FISH), was unsuccessful. Both probes we used, (TTAGG)_*n*_ and (TTAGGGATGG)*_n_*, failed to hybridize with the telomeres of *G.dispar*, indicating the presence of a different telomeric motif in this species. To address this issue, we performed FISH on *G.dispar* chromosomes using as a probe the DNA sequence (TTAGGGGTGG)*_n_*, which is the second most frequent telomeric sequence in Pentatomomorpha.

## ﻿Material and methods

Adult males of *G.dispar* were collected in the Voronezh Province, Russia (51°49'N, 39°23'E), in August 2023. Specimens were fixed in a medium consisting of 96% ethanol and glacial acetic acid (3:1) and stored in the fixative at 4 °C until further use. Chromosome preparations were obtained from testes using the squash method and were made permanent using the dry-ice technique (Conger and Fairchild 1953). The Schiff-Giemsa method described by Grozeva and Nokkala (1996) was used for standard karyotype examination. The FISH procedure was performed according to the protocol described by Kuznetsova et al. (2025). The DNA sequence (TTAGGGGTGG)*_n_* was used as a probe for FISH. This sequence was amplified and labeled with Biotin-16-dUTP by PCR without a DNA template, using the primer 5’-TAACCACCCCTAACCACCCCTAA-3’/5’- GGTTAGGGGTGGTTAGGGGTGG-3’.

Fluorescence images were acquired using a Leica DFC 345 FX camera mounted on a Leica DM 6000B microscope equipped with a 100x objective lens. The images were processed using the Leica Application Suite version 4.5.0 software, which includes the Image Overlay module.

## ﻿Results and discussion

**Figure 1.**
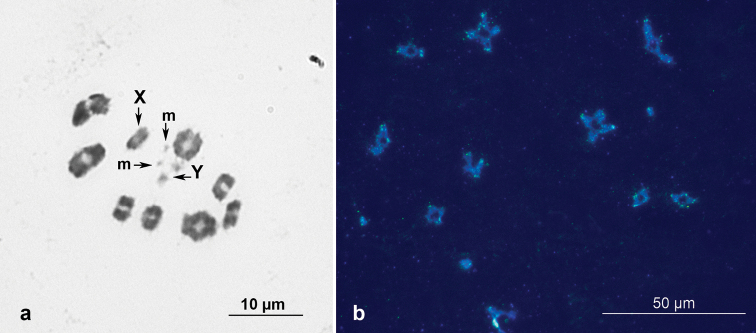
Meiotic karyotype (diakinesis stage) of *G.dispar* after Shiff-Giemsa staining (**a**) and after FISH with the (TTAGGGGTGG)*_n_* telomeric probe (**b**). Bright green signals at the ends of the chromosomes (**b**) indicate the presence of the TTAGGGGTGG motif. Scale bars: 10 μm (**a**); 50 μm (**b**).

*Geocorisdispar* males possess a chromosome number of 2n = 20 (18 + XY) with a pair of microchromosomes (m-chromosomes), and a meioformula of n = 8AA + mm + X + Y (Fig. 1a). This is consistent with our previously published data on the karyotype of this species (Kuznetsova et al. 2025). FISH with the probe (TTAGGGGTGG)*_n_* produced distinct hybridization signals at the ends of the chromosomes (Fig. 1b), indicating the presence of a 10-base pair telomeric motif, TTAGGGGTGG.

The worldwide family Geocoridae, to which *G.dispar* belongs, comprises five subfamilies, 37 genera and 321 species. More than three-quarters of the species belong to the subfamily Geocorinae, with nearly half (150 species) belonging to the genus *Geocoris* Fallén, 1814 (Dellapé and Henry 2020). Geocoridae are classified within the superfamily Lygaeoidea in the infraorder Pentatomomorpha (Liu et al. 2019; Weirauch et al. 2019; Zhao et al. 2024). Our finding of the telomeric structure in *G.dispar* represents the first data for Geocoridae, showing the presence of a TTAGGGGTGG motif, which was not previously known for Lygaeoidea as a whole. In contrast, three previously studied Lygaeoidea species, *Kleidocerysresedae* (Panzer, 1797) and *Lygaeusequestris* (Linnaeus, 1758) from the family Lygaeidae, and *Dimorphopterusspinolae* (Signoret, 1857) from the family Blissidae, have a telomeric motif TTAGGGATGG. Note that this latter is more frequent in Pentatomomorpha and Cimicomorpha and is considered a candidate ancestral motif for the clade Terheteroptera in general (Kuznetsova et al. 2025).

**Figure 2.**
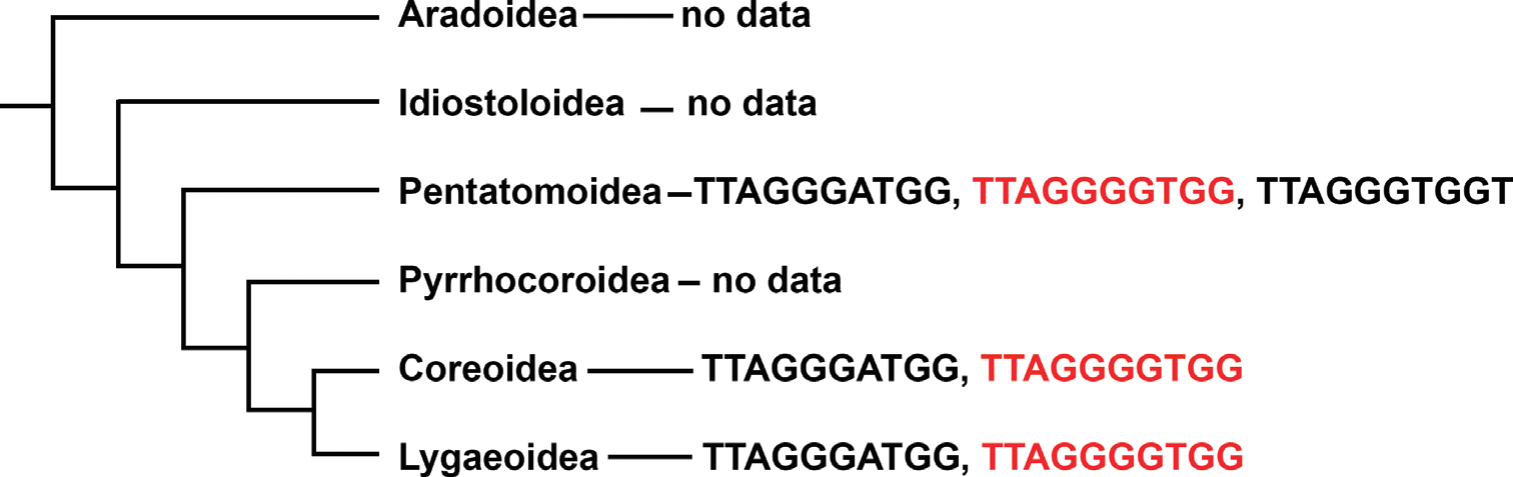
Telomeric repeats found in three superfamilies of Pentatomomorpha mapped onto the phylogenetic tree (according to Weirauch et al. 2019).

Pentatomomorpha is the second largest infraorder of true bugs (after Cimicomorpha), comprising over 14,000 species belonging to approximately 40 families within six superfamilies: Aradoidea, Pentatomoidea, Coreoidea, Lygaeoidea, Pyrrhocoroidea and Idiostoloidea (Liu et al. 2019; Weirauch et al. 2019). In the three superfamilies for which data are available (Pentatomoidea, Coreoidea and Lygaeoidea), no species were found to have the canonical 5-letter “insect type” telomeric motif. Instead, three variants of the 10-bp telomeric motif, including TTAGGGATGG, TTAGGGTGGT and TTAGGGGTGG, were identified based on FISH or chromosome-level genome assembly analysis (Lukhtanov and Pazhenkova 2023; Kuznetsova et al. 2025; present study). All three motifs were found in Pentatomoidea: TTAGGGATGG and TTAGGGGTGG in Pentatomidae, and TTAGGGTGGT in Acanthosomatidae. Two motifs, TTAGGGATGG and TTAGGGGTGG, were revealed in the sister superfamilies Coreoidea and Lygaeoidea. The first motif is present in the families Coreidae and Rhopalidae (Coreoidea), as well as in the families Lygaeidae and Blissidae (Lygaeoidea). The second motif is present in the families Coreidae and Alydidae (Coreoidea) and in the family Geocoridae (Lygaeoidea) (Fig. 2).

Accepting the hypothesis that the TTAGGGATGG motif in Terheteroptera is ancestral (Kuznetsova et al. 2025), we must recognize that the other two 10-nucleotide motifs found in Pentatomomorpha emerged as a result of the substitution of one nucleotide (in the TTAGGGGTGG motif) or two nucleotides (in the TTAGGGTGGT motif) during the evolution of this infraorder. These substitutions probably occurred relatively easily, resulting in considerable diversity of telomeric sequences in Pentatomomorpha. The TTAGGGGTGG motif appears to have arisen independently during the evolution of the superfamilies Pentatomoidea, Coreoidea and Lygaeoidea. However, data on this topic remain limited, and further studies are needed.
